# Enzymatic activity of a subtilisin homolog, Tk-SP, from *Thermococcus kodakarensis* in detergents and its ability to degrade the abnormal prion protein

**DOI:** 10.1186/1472-6750-13-19

**Published:** 2013-02-28

**Authors:** Azumi Hirata, Yuki Hori, Yuichi Koga, Jun Okada, Akikazu Sakudo, Kazuyoshi Ikuta, Shigenori Kanaya, Kazufumi Takano

**Affiliations:** 1 Laboratory of Biological Chemistry, Department of Biomolecular Chemistry, Kyoto Prefectural University, Kyoto, Japan; 2 Department of Material and Life Science, Graduate School of Engineering, Osaka University, Suita, Japan; 3 Faculty of Medicine, University of the Ryukyus, Nishihara, Japan; 4 Department of Virology, Center for Infectious Disease Control, Research Institute for Microbial Diseases, Osaka University, Suita, Japan

**Keywords:** Serine protease, Hyperthermophilic archaeon, Subtilisin, Detergent compatibility, Prion, Transmissible spongiform encephalopathies (TSE), Degradation, Decontamination

## Abstract

**Background:**

Tk-SP is a member of subtilisin-like serine proteases from a hyperthermophilic archaeon *Thermococcus kodakarensis*. It has been known that the hyper-stable protease, Tk-SP, could exhibit enzymatic activity even at high temperature and in the presence of chemical denaturants. In this work, the enzymatic activity of Tk-SP was measured in the presence of detergents and EDTA. In addition, we focused to demonstrate that Tk-SP could degrade the abnormal prion protein (PrP^Sc^), a protease-resistant isoform of normal prion protein (PrP^C^).

**Results:**

Tk-SP was observed to maintain its proteolytic activity with nonionic surfactants and EDTA at 80°C. We optimized the condition in which Tk-SP functions efficiently, and demonstrated that the enzyme is highly stable in the presence of 0.05% (w/v) nonionic surfactants and 0.01% (w/v) EDTA, retaining up to 80% of its activity. Additionally, we also found that Tk-SP can degrade PrP^Sc^ to a level undetectable by western-blot analysis.

**Conclusions:**

Our results indicate that Tk-SP has a great potential for technological applications, such as thermo-stable detergent additives. In addition, it is also suggested that Tk-SP-containing detergents can be developed to decrease the secondary infection risks of transmissible spongiform encephalopathies (TSE).

## Background

Proteases form a major part of the industrial enzyme market, and are applied to detergents as well as food, leather, and fabric processing, and are also used as catalysts in organic synthesis and as therapeutics [1]. With the annual protease sales of about $1.5–1.8 billion, these enzymes account for 60% of the total enzyme market. Detergent proteases, particularly alkaline proteases, with an annual market of about $1 billion, account for the largest part of protease application. Subtilisin Carlsberg and related subtilisin serine proteases represent the first generation of detergent proteases with an optimal pH of 9–10. The second-generation detergent proteases, having a higher optimal pH of 10–11 and greater temperature stability, are produced from alkalophilic strains of *Bacillus*. The third generation consists of detergent proteases in which the active sites have been modified or various attempts have been made to enhance their stability through site-directed mutagenesis and protein engineering [2,3].

Proteolytic enzymes from microorganisms are the most widely exploited enzymes in the detergent industries [4,5]. These enzymes are incorporated into detergent formulations to obtain certain characteristics, such as activity and stability in high alkaline pH range and high temperatures, compatibility with compounds used in detergents such as surfactants, perfumes, and bleaches, and hydrolysis specificity towards other proteins [6]. Therefore, there is always a need for newer hyper-stable alkaline proteases with potential application in detergent formulations.

More recently, studies on enzymatic degradation of prion protein have highlighted the ability of some proteases to digest this class of protein [7-9]. Prion diseases are fatal neurodegenerative disorders that include Creutzfeldt-Jacob disease (CJD), Gerstmann-Sträussler-Scheinker syndrome, fatal familial insomnia, and kuru in humans, bovine spongiform encephalopathy (BSE) in cattle, and scrapie in sheep [10,11]. Their central feature is the posttranslational conversion of host-encoded, cellular prion protein (PrP^C^) to an abnormal isoform, known as PrP^Sc^. This transition appears to involve only a conformational change to a predominantly β-pleated structure, and confers PrP^Sc^ with partial resistance to proteolytic degradation and detergent insolubility [10-12]. PrP^Sc^ is largely unaffected by standard methods of sterilization; thus, contaminated neurosurgical instruments are the major cause of human transmissible spongiform encephalopathies (TSEs), in addition to exposure to infectious materials through the use of human cadaveric-derived pituitary hormones as well as dural and cornea homotransplants [13]. The results of numerous studies designed to define conditions for the inactivation of PrP^Sc^ concluded that protein denaturants, such as sodium dodecyl sulfate (SDS) [13,14], are effective in reducing infectivity, but that complete inactivation requires extremely intense conditions, such as 1 h of autoclaving at 134°C or treatment with 2 N NaOH or sodium hypochlorite for 1 h [12]. However, such intense conditions cannot be applied to certain equipments such as endoscope. Hence, validated methods to inactivate PrP^Sc^ are highly desirable.

*Thermococcus kodakarensis* is a hyperthermophilic archaeon, which grows most optimally at 90°C [15]. Its genome contains three genes encoding subtilisin-like serine proteases: Tk-subtilisin (GenBank: YP184088) [16], Tk-SP (GenBank: YP184102) [17], and Tk-0076 (GenBank: YP182489). Of these homologs, Tk-subtilisin and Tk-SP have been structurally and functionally well studied [16-27]. Tk-subtilisin and Tk-SP are highly thermostable enzymes with optimal temperature for activity in the range of 90–100°C and show broad substrate specificity. Tk-subtilisin and Tk-SP exhibit the pH dependence and temperature dependence of the activities. Tk-SP exhibits 10 to 70% of the maximal activity at a neutral pH range between 6.0 and 8.0 at 20°C. It exhibits 10 to 50% of the maximal activity at between 30 and 60°C [17]. In particular, Tk-SP is highly resistant to heat, 5% SDS, 8 M urea, 10% Triton X-100, or 10 mM EDTA; however, unlike Tk-subtilisin and bacterial subtilisin, Tk-SP does not require Ca^2+^ for folding [17,25]. Thus, Tk-SP has a great potential for technological applications.

In the present study, to examine the activity of Tk-SP in the presence of 12 surfactants (four nonionic detergents, three anionic detergents, two cationic detergents, and three amphionic detergents) and EDTA at high temperatures, Tk-SP was overproduced in *E*. *coli* and purified, and subsequently analyzed for its relative activity at 80°C and 90°C using azocasein as the substrate. We found that Tk-SP could maintain its proteolytic activity in the presence of surfactants, suggesting that this enzyme could be a candidate for industrial applications in detergent formulation.

In addition, to determine whether Tk-SP is capable of degrading PrP^Sc^, PrP^Sc^ (Chandler strain) accumulated in scrapie-infected mouse brain homogenate (MBH) was incubated with Tk-SP and then detected by western-blot analysis. We observed that Tk-SP can disrupt PrP^Sc^ to a level undetectable by western-blot analysis, suggesting that this enzyme has a remarkable characteristic, considering its potential application for the inactivation of PrP^Sc^.

## Results and discussion

### Effect of surfactants and EDTA on Tk-SP activity

**Figure 1:**
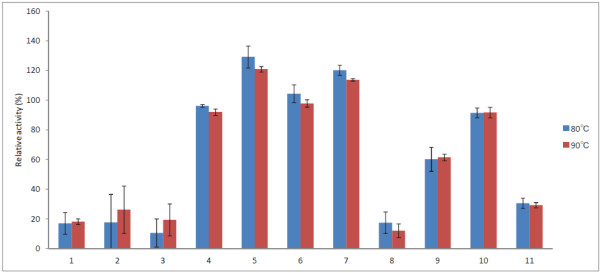
**Effect of 0.1% (w/v) surfactants on the activity of Tk-SP.** Tk-SP was incubated at 80°C or 90°C for 20 min in 50 mM Tris–HCl (pH 7.5) containing a 0.1% (w/v) surfactant, and the relative activities were determined using azocasein as the substrate. Each experiment was carried out three times, and the average values are shown together with the error bars. Lane 1, EMAL TD; lane 2, EMAL 20C; lane 3, SDS; lane 4, EMULGEN 147; lane 5, EMULGEN LS-114; lane 6, EMULGEN PP-290; lane 7, RHEODOL Tw-0120 V; lane 8, SANISOL C; lane 9, AMPHITOL 24B; lane 10, AMPHITOL 20Y-B; lane 11, AMPHITOL 20 N.

**Figure 2:**
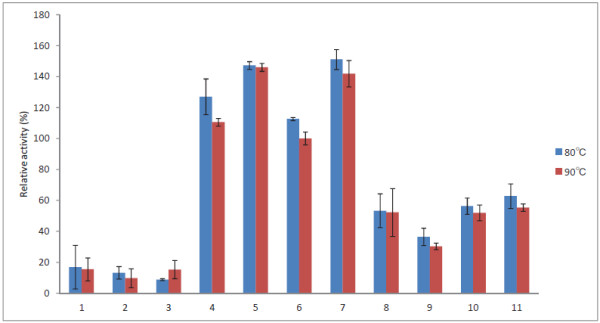
**Effect of 1% (w/v) surfactants on the activity of Tk-SP.** Tk-SP was incubated at 80°C or 90°C for 20 min in 50 mM Tris–HCl (pH 7.5) containing a 1% (w/v) surfactant, and the relative activities were determined using azocasein as the substrate. Each experiment was carried out three times, and the average values are shown together with the error bars. Lane 1, EMAL TD; lane 2, EMAL 20C; lane 3, SDS; lane 4, EMULGEN 147; lane 5, EMULGEN LS-114; lane 6, EMULGEN PP-290; lane 7, RHEODOL Tw-0120 V; lane 8, SANISOL C; lane 9, AMPHITOL 24B; lane 10, AMPHITOL 20Y-B; lane 11, AMPHITOL 20 N.

Some studies have suggested that an effective detergent protease must be compatible and stable with commonly used detergent compounds, such as surfactants, which might be present in the formulation [28,29]. To analyze the effects of surfactants on the stability and enzymatic activity of Tk-SP, 100 nM Tk-SP was incubated in 50 mM Tris–HCl (pH 7.5) containing 0.1 or 1% (w/v) surfactants for 20 min at 80°C or 90°C using azocasein as the substrate. While Tk-SP is known to exhibit its highest activity at 100°C (510 ± 50 U/mg), its relative activities at 90°C or 80°C are 95 and 80%, respectively [17]. One unit of enzymatic activity is defined as the amount of enzyme that increases the A_440_ value of the assay reaction mixture by 0.1 in 1 min [17]. The results of our analysis are summarized in Figures 1 and 2. The data show that Tk-SP retained its activity in the presence of surfactants tested at 80°C and 90°C. Furthermore, Tk-SP was found to be highly stable in the presence of both 0.1 and 1% (w/v) nonionic surfactants. Particularly, Tk-SP retained more than 100% of its activity in the presence of four of the nonionic surfactants, namely, EMULGEN 147, EMULGEN LS-114, EMULGEN PP-290, and RHEODOL Tw-0120 V. However, the color of the reaction mixture changed to white after the addition of the cationic surfactant, QUARTAMIN 60 W, indicating that Tk-SP was less stable in the presence of QUARTAMIN 60 W. Furthermore, although Tk-SP was stable in the presence of 0.1% (w/v) AMPHITOL 20Y-B, the presence of 1% (w/v) AMPHITOL 20Y-B caused strong inhibitions, resulting in Tk-SP retaining only 50% of its activity. In contrast, the activities in the presence of 1% SANISOL C and AMPHITOL 20 N were higher than those at the concentration of 0.1%. This may relate to the critical micelle concentration of the surfactants.

In the presence of anionic surfactants, Tk-SP was unstable, losing up to 80% of its activity. On the other hand, a previous study analyzed the activity of Tk-SP at 55°C using *N*-succinyl-Ala-Ala-Pro-Phe-*p*-nitroanilide (Suc-AAPF-*p*NA) as the substrate, and found that the enzyme is highly resistant to 5% (w/v) SDS [17]. This discrepancy in the findings may be due to the temperature of the reaction. In addition, the concentration of Tk-SP analyzed in the previous study was ten-fold higher than that in this study, suggesting that high concentration of Tk-SP may increase the stability. Thus, from these results, it can be concluded that Tk-SP is more stable in the presence of nonionic surfactants, suggesting that intramolecular interactions of ion pairs of Tk-SP may contribute to its stability. The improvement in the activity of Tk-SP in the presence of nonionic surfactants might be due to the stimulation of conformational changes in Tk-SP or the substrate, leading to activity gain.

**Figure 3:**
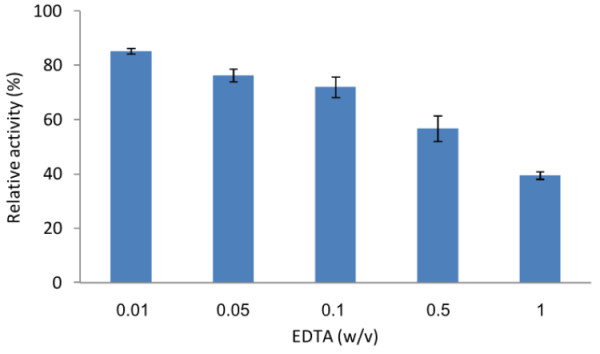
**Effect of EDTA on the activity of Tk-SP.** Tk-SP was incubated at 80°C for 20 min in 50 mM Tris–HCl (pH 7.5) containing EDTA at the concentration indicated, and the relative activities were determined using azocasein as the substrate. Each experiment was carried out three times, and the average values are shown together with the error bars.

Chelating agents, such as EDTA, which function as water softeners and assist in the removal of stains, are valuable components of most of the detergents [28,29]. As Tk-SP has been found to exhibit activity at 80°C in the presence of 0.37% (w/v) EDTA [17], to analyze the stability and activity of Tk-SP against different concentrations of EDTA, 100 nM Tk-SP was incubated in 50 mM Tris–HCl (pH 7.5) containing 0.01, 0.05, 0.1, 0.5, or 1% (w/v) EDTA for 20 min at 80°C using azocasein as the substrate. As can be seen in Figure 3, the Tk-SP activity decreased with the increasing concentration of EDTA. Several reports have shown that the active structure of serine proteases contains Ca^2+^-binding site(s), and that the removal of Ca^2+^ from the strong binding site is associated with a significant reduction in thermal stability [30]. Our results are in agreement with the previous findings showing that two Ca^2+^ ions bind to the β-jelly roll domain of Tk-SP [24] and that Tk-SP requires Ca^2+^ ions for maximal stability [17]. In the present study, Tk-SP retained 80% of its activity in the presence of 0.01% (w/v) EDTA (Figure 3), suggesting that this enzyme will be effective in the presence of 0.01% (w/v) EDTA, which is a commonly used concentration of EDTA in detergents.

**Figure 4:**
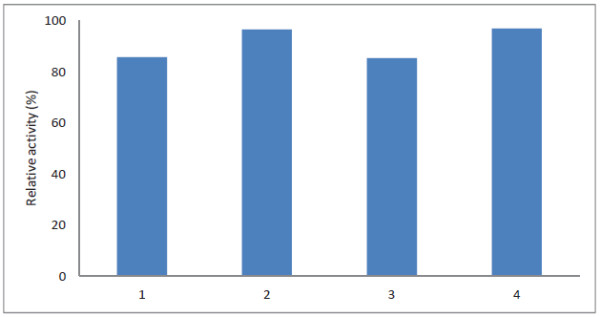
**Effect of 0.05% (w/v) nonionic surfactants and 0.01% (w/v) EDTA on the activity of Tk-SP.** Tk-SP was incubated at 80°C for 20 min in 50 mM Tris–HCl (pH 7.5) containing the nonionic surfactants indicated, and the relative activities were determined using azocasein as the substrate. Each experiment was carried out three times, and the average values are shown together with the error bars. Lane 1, EMULGEN 147; lane 2, EMULGEN LS-114; lane 3, EMULGEN PP-290; lane 4, RHEODOL Tw-0120 V.

To analyze the compatibility and activity of Tk-SP in the presence of both surfactants and EDTA, the enzyme was incubated in the presence of four 0.05% (w/v) nonionic surfactants and 0.01% (w/v) EDTA at 80°C. As shown in Figure 4, Tk-SP was highly stable in the presence of all of the nonionic surfactants, retaining up to 80% of its activity. Among the additives examined, the best result with respect to Tk-SP retaining almost its entire initial activity was observed in the presence of EMULGEN LS-114 and RHEODOL Tw-0120 V. Previous reports have shown that stability of protease could be achieved either through site-directed mutagenesis or protein engineering; however, the protease, Tk-SP, examined in the present study has been noted to show inherent stability in the presence of surfactants and EDTA. In addition, Tk-SP has also been observed to exhibit activity at high temperature, suggesting that this enzyme meets the standard requirements of the washer-disinfector (ISO 15883), which recommend that a suitable temperature of thermal disinfection for medical instruments should be in the range of 80 ~ 95°C [31]. Considering the above-mentioned characteristics, it can be concluded that Tk-SP is a potential candidate for industrial applications.

### Digestion of infectious MBH with Tk-SP

**Figure 5:**
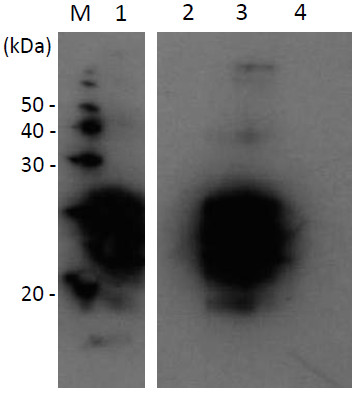
**Western blots of PrP**^**Sc **^**Tk-SP digests.** 1% MBH was subjected to digestion at 100°C for 1 h with buffer only (lane 1), 0.02 mg/ml (0.4 mM) Tk-SP (lane 2), 1% SDS (lane 3), or 0.02 mg/ml (0.4 mM) Tk-SP plus 1% SDS (lane 4). The treated samples were analyzed by western blot using mAb SAF-83. Lane M, marker.

Our western-blot analysis revealed that Tk-SP can reduce the levels of SAF-83 immunoreactive material (Figure 5). The western blots probed with anti-prion antibody, SAF-83, showed three characteristic anti-prion antibody reactive bands corresponding to the unglycosylated, monoglycosylated, and diglycosylated forms of PrP^Sc^. We found that 1% (w/v) SDS was relatively poor at eliminating PrP^Sc^ with little or no effect, while 0.02 mg/ml (approximately 0.4 mM) Tk-SP produced complete digestion. Furthermore, a combination of 0.02 mg/ml (0.4 mM) Tk-SP and 1% (w/v) SDS was noted to digest infectious MBH, suggesting that Tk-SP has the ability to digest PrP^Sc^ in the absence or presence of SDS. SDS is known to be a strong denaturant of proteins. It has the ability to unfold most of the proteins through the interaction between the charged head group of SDS and the positively charged amino acid side chains of proteins, and that between the alkyl chain of SDS and the nonpolar parts on the surface as well as in the interior of proteins [32]. As SDS-stable enzymes have been rarely reported, it is important to remark the capability of Tk-SP to inactivate PrP^Sc^, as observed in this study, and its ability to retain only 20% of its activity in the presence of SDS (Figures 1 and 2). Our results also suggest that Tk-SP may have potential application as a detergent additive to decrease the infectivity of PrP^Sc^. Here, the concentration of Tk-SP (0.02 mg/ml; 0.4 mM) in Figure 5 was higher than that (100 nM) of Figures 1 and 2. To determine the minimum amount of Tk-SP for the complete digestion of prion protein in this condition, further quantitative analysis is necessary.

Previous reports have shown that alkaline pH is critical for effective digestion of PrP^Sc^ by subtilisin-type proteases; at pH 12, stable β-sheet structures may be significantly reduced or eliminated, allowing greater access or susceptibility to proteases [7,8]. The ability of the enzymes to inactivate prions requires them to function at pH 12, a condition in which the conformation is accessible and susceptible to the protease. Tk-SP has been found to exhibit high activity at a wide range of pH between 7.0 and 11.5; additionally, the enzyme has been noted to be stable even at a pH of 12 [17]. In the present study, digestion of infectious MBH was assessed at a pH of 8.0, and Tk-SP showed complete inactivation of PrP^Sc^, suggesting that the enzyme can be useful for the inactivation of PrP^Sc^ not only in alkaline pH, but also in neutral pH. Thus, the remarkable characteristic of the ability of Tk-SP to degrade PrP^Sc^ completely indicates its potential application for the digestion of PrP^Sc^.

## Conclusions

In the present study, Tk-SP, a subtilisin homolog from *Thermococcus kodakarensis*, exhibited more than 100% of its activity and was highly stable in the presence of 0.1% (w/v) nonionic surfactants, but lost up to 20% of its activity in the presence of 0.01% (w/v) EDTA at 80°C. However, in the presence of 0.05% (w/v) nonionic surfactants, such as EMULGEN LS-114 or RHEODOL Tw-0120 V, and 0.01% (w/v) EDTA, Tk-SP retained almost its entire initial activity. Furthermore, the enzyme showed inherent stability in the presence of both surfactants and EDTA without site-directed mutagenesis or protein engineering. Moreover, Tk-SP was able to reduce the level of PrP^Sc^ as detected by western-blot analysis. Thus, Tk-SP can possibly be used as a detergent adjunct for industrial and healthcare applications.

## Methods

### Protein preparation

The pET25b derivative for the overproduction of Pro-Tk-SP (Met + Ala1–Gly640) was constructed previously [17]. *E*. *coli* BL21-codonPlus (DE3) was transformed with the pET25b derivative for the overproduction of Tk-SP. Pro-Tk-SP was processed into Tk-SP, a 44-kDa protein (Val114–Val539) during the maturation process, in which N-propeptide (Ala1–Ala113) is autoprocessed first, and then, the C-domain (Asp540–Gly640) is removed [14,24,25]. For overproduction, these transformants were grown at 37°C in NZCYM medium (Novagen) containing 50 μg/ml ampicillin and 35 μg/ml chloramphenicol. When the absorbance of 600 nm of the culture reached around 0.5, 1.0 mM isopropyl-β-D-thiogalactopyranoside (IPTG) was added to the culture medium and cultivation was continued for an additional 4 h. The cells were then harvested by centrifugation at 6000 × g for 10 min at 4°C and subjected to the following purification procedures at 4°C.

The cells were suspended in 20 mM Tris–HCl (pH 9.0), disrupted by sonication lysis, and centrifuged at 30,000 × g for 30 min at 4°C. The soluble fraction was collected and centrifuged at 30,000 × g for 30 min at 4°C to remove the precipitates. The protein was precipitated to 30% saturation by adding ammonium sulfate to the resultant supernatant. The pellet was collected by centrifugation at 30,000 × g for 30 min and dissolved in 20 mM Tris–HCl (pH 7.5). The resultant solution was dialyzed against 20 mM Tris–HCl (pH 7.5) and applied to a HiTrapQ HP column (GE Healthcare) equilibrated with the same buffer. The protein was eluted from the column by linearly increasing the NaCl concentration from 0 to 1.0 M. The fractions containing the protein were collected, dialyzed against 20 mM Tris–HCl (pH 7.5), concentrated using the Centricon (Millipore) ultrafiltration system, incubated at 80°C for 2 h, and stored at −20°C until further use.

The purity of the protein was analyzed by SDS-Polyacrylamide Gel Electrophoresis (SDS-PAGE) using a 12% polyacrylamide gel, followed by staining with Coomassie Brilliant Blue (CBB). For these analyses, the sample was prepared by precipitating the protein using 1% (w/v) trichloroacetic acid (TCA), dissolving the pellet in SDS sample buffer (50 mM Tris–HCl (pH 6.8), 0.1 M dithiothreitol, 2% SDS, 10% (v/v) glycerol, and 0.005% (w/v) bromophenol blue), neutralizing the pH of the resultant solution, and boiling it for 5 min. The protein concentration was determined by UV absorption using a cell with an optical path length of 1 cm and an A_280_ value of 1.83 for 0.1% (1 mg/ml) Tk-SP solution. This value was calculated by using ε = 1526 and 5225/M/cm for Tyr and Trp, respectively, at 280 nm [25].

### Enzymatic activity

The enzymatic activity was determined by using azocasein (Sigma Chemical Co.) as the substrate at 80°C or 90°C. The reaction mixture (540 μl) contained 50 mM Tris–HCl (pH 7.5) and 0.2% (w/v) azocasein. Prior to the addition of Tk-SP, the reaction mixture was preincubated at the respective temperatures for 5 min. The enzymatic reaction was initiated by adding 100 nM Tk-SP and terminated by the addition of 400 μl of 1.5% (w/v) TCA. The reaction time was 20 min. Immediately after incubation, the samples were cooled at 4°C for 10 min. After centrifugation at 15,000 × g for 15 min at 4°C, an aliquot of the supernatant (640 μl) was withdrawn, mixed with 80 μl of 2 M NaOH, and measured for absorption at 440 nm (A_440_). The solubility of digested products of azocasein in the presence of detergents did not affect the activity assay in this condition. One unit of enzymatic activity is defined as the amount of enzyme that increases the A_440_ value of the assay reaction mixture by 0.1 in 1 min.

### Effect of surfactants and EDTA on protease activity

To analyze the effects of surfactants and EDTA on the activity of Tk-SP, the enzyme’s activities were measured at 80°C or 90°C using azocasein as the substrate in the presence or absence of various concentrations of surfactants – four nonionic surfactants (EMULGEN 147, EMULGEN LS-114, EMULGEN PP-290, and RHEODOL Tw-0120 V; Kao Corporation, Tokyo, Japan), three anionic surfactants (EMAL TD, EMAL 20C (Kao), and SDS (Nacalai Tesque, Inc., Kyoto, Japan)), two cationic surfactants (QUARTAMIN 60 W and SANISOL C; Kao), and three amphionic surfactants (AMPHITOL 24B, AMPHITOL 20Y-B, and AMPHITOL 20 N; Kao) – and/or various concentrations of EDTA. The enzyme activity of a control without any surfactants and/or EDTA, incubated under similar conditions, was taken as 100%.

### Enzymatic digestion of infectious MBH

Infectious 1% MBH in 200 mM Tris–HCl buffer (pH 8.0) was digested with 0.02 mg/ml (0.4 mM) Tk-SP in 0.5 M Tris–HCl buffer, and adjusted to pH 8.0 at 100°C for 1 h in the presence or absence of 1% SDS. Tk-SP was inactivated by adding 50 mM diisopropylfluorophosphate (DFP) to the sample. Positive control material at pH 8.0 was prepared in the same way, but in the absence of Tk-SP and SDS. SDS-PAGE was performed using 15% polyacrylamide gel. The samples were electrophoresed and then transferred to nitrocellulose membrane at constant amperage of 140 mA for 90 min. The membrane was immersed into 5% skim milk in 10 mM Tris-buffered saline (TBS) for 12 h to block nonspecific binding and washed with TBS containing 0.1% Tween 20. The membrane was then incubated with mouse anti-Prion protein monoclonal antibody (SAF-83) (Cayman Chemical Company; MI) diluted at a ratio of 1:1000 for 1 h at room temperature, followed by incubation with horseradish peroxidase-conjugated anti-mouse IgG (Sigma; St Louis, MO) for 1 h at room temperature. Immunoreactivity was visualized using ECL Plus Western Blotting Detection Reagents (GE Healthcare), according to the manufacturer’s instructions.

## Competing interests

There are no financial or non-financial competing interests relating to the publication of this manuscript.

## Authors’ contributions

KT and YK conceived and supervised the experiments. AH, YH, YK, and JO performed the experiments. AS, KI, and SK contributed the reagents, materials, and analysis tools. AH wrote the paper. YK, AS, and KT helped in interpretation of data and discussion of results. All the authors have read and approved the final manuscript.

## References

[B1] Zhu D Wu Q Wang N Muray M-Y Industrial enzymes Comprehensive Biotechnology. Volume 3 2011 2 Waltham: Elsevier 3 13

[B2] Bryan PN Protein engineering of subtilisin Biochim Biophys Acta 2000 1543 203 222 10.1016/S0167-4838(00)00235-1 11150607

[B3] Ward OP Muray M-Y Proteases Comprehensive Biotechnology. Volume 3 2011 2 Waltham: Elsevier 571 582

[B4] Kirk O Borchert TV Fuglsang CC Industrial enzyme applications Curr Opin Biotechnol 2002 13 345 351 10.1016/S0958-1669(02)00328-2 12323357

[B5] Gessesse A Hatti-Kaul R Gashe BA Mattiasson B Novel alkaline proteases from alkaliphilic bacteria grown on chicken feather Enzyme Microb Technol 2003 32 519 524 10.1016/S0141-0229(02)00324-1

[B6] Sellami-Kamoun A Haddar A Ali NE Ghorbel-Frikha B Kanoun S Nasri M Stability of thermostable alkaline protease from *Bacillus licheniformis* RP1 in commercial solid laundry detergent formulations Microbiol Res 2008 163 299 306 10.1016/j.micres.2006.06.001 16872818

[B7] Mcleod AH Murdoch H Dickinson J Dennis MJ Hall GA Buswell CM Carr J Taylor DM Sutton JM Raven NDH Proteolytic inactivation of the bovine spongiform encephalopathy agent Biochem Biophys Res Commun 2004 317 1165 1170 10.1016/j.bbrc.2004.03.168 15094392

[B8] Dickinson J Murdoch H Dennis MJ Hall GA Bott R Crabb WD Penet C Sutton JM Raven NDH Decontamination of prion protein (BSE301V) using a genetically engineered protease J Hosp Infect 2009 72 65 70 10.1016/j.jhin.2008.12.007 19201054

[B9] Langeveld JPM Wang JJ Van de Wiel DFM Shih GC Garssen GJ Bossers A Shih JCH Enzymatic degradation of prion protein in brain stem from infected cattle and sheep J Infect Dis 2003 188 1782 1789 10.1086/379664 14639552

[B10] Prusiner SB Prions Proc Natl Acad Sci USA 1998 95 13363 13383 10.1073/pnas.95.23.13363 9811807 PMC33918

[B11] Wissmann C The state of the prion Nat Rev Microbiol 2004 2 861 871 10.1038/nrmicro1025 15494743

[B12] Collinge J Prion diseases of humans and animals: Their causes and molecular basis Annu Rev Neurosci 2001 24 519 550 10.1146/annurev.neuro.24.1.519 11283320

[B13] WHO Infection Control Guidelines for Transmissible Spongiform Encephalopathieshttp://www.who.int/csr/resources/publications/bse/whocdscsraph2003.pdf

[B14] Peretz D Supattapone S Giles K Vergara J Freyman Y Lessard P Safar JG Glidden DV McCulloch C Nguyen HOB Scott M DeArmond SJ Prusiner SB Inactivation of prions by acidic sodium dodecyl sulfate J Virol 2006 80 322 331 10.1128/JVI.80.1.322-331.2006 16352557 PMC1317507

[B15] Atomi H Fukui T Kanai T Morikawa M Imanaka T Description of Thermococcus kodakaraensis sp. nov., a well studied hyperthermophilic archaeon previously reported as Pyrococcus sp. KOD1 Archaea 2004 1 263 267 10.1155/2004/204953 15810436 PMC2685570

[B16] Pulido M Saito K Tanaka S Koga Y Morikawa M Takano K Kanaya S **Ca**^**2+**^**-dependent maturation of subtilisin from a hyperthermophilic archaeon, *****Thermococcus kodakaraensis*****: the propeptide is a potent inhibitor of the mature domain but is not required for its folding.** Appl Environ Microbiol 2006 72 4154 4162 10.1128/AEM.02696-05 16751527 PMC1489632

[B17] Foophow T Tanaka S Koga Y Takano K Kanaya S Subtilisin-like serine protease from hyperthermophilic archaeon *Thermococcus kodakaraensis* with N- and C-terminal propeptides Protein Eng Des Sel 2010 23 347 355 10.1093/protein/gzp092 20100702

[B18] Tanaka S Saito K Chon H Matsumura H Koga Y Takano K Kanaya S Crystal structure of unautoprocessed precursor of subtilisin from a hyperthermophilic archaeon J Biol Chem 2007 282 8246 8255 10.1074/jbc.M610137200 17237225

[B19] Tanaka S Matsumura H Koga Y Takano K Kanaya S Four new crystal structures of Tk-subtilisin in unautoprocessed, autoprocessed and mature forms: Insight into structural changes during maturation J Mol Biol 2007 372 1055 1069 10.1016/j.jmb.2007.07.027 17706669

[B20] Pulido MA Koga Y Takano K Kanaya S Directed evolution of Tk-subtilisin from a hyperthermophilic archaeon: identification of a single amino acid substitution responsible for low-temperature adaptation Protein Eng Des Sel 2007 20 143 153 10.1093/protein/gzm006 17351019

[B21] Pulido MA Tanaka S Sringiew C You DJ Matsumura H Koga Y Takano K Kanaya S Requirement of left-handed Glycine residue for high stability of the Tk-subtilisin propeptide as revealed by mutational and crystallographic analyses J Mol Biol 2007 374 1359 1373 10.1016/j.jmb.2007.10.030 17988685

[B22] Tanaka S Takeuchi Y Matsumura H Koga Y Takano K Kanaya S Crystal structure of Tk-subtilisin folded without propeptide: Requirement of propeptide for acceleration of folding FEBS Lett 2008 582 3875 3878 10.1016/j.febslet.2008.10.025 18951896

[B23] Tanaka S Matsumura H Koga Y Takano K Kanaya S Identification of the interactions critical for propeptide-catalyzed folding of Tk-Subtilisin J Mol Biol 2009 394 306 319 10.1016/j.jmb.2009.09.028 19766655

[B24] Foophow T Tanaka S Angkawidjaja C Koga Y Takano K Kanaya S **Crystal structure of a subtilisin homologue, Tk-SP, from *****Thermococcus kodakaraensis:*** Requirement of a C-terminal β-jelly roll domain for hyperstability J Mol Biol 2010 400 865 877 10.1016/j.jmb.2010.05.064 20595040

[B25] Sinsereekul N Foophow T Yamanouchi M Koga Y Takano K Kanaya S An alternative mature form of subtilisin homologue, Tk-SP, from *Thermococcus kodakaraensis* identified in the presence of Ca^2+^ FEBS Lett 2011 278 1901 1911 10.1111/j.1742-4658.2011.08107.x 21443525

[B26] Kannan Y Koga Y Inoue Y Haruki M Takagi M Imanaka T Morikawa M Kanaya S Active subtilisin-like protease from a hyperthermophilic archaeon in a form with a putative prosequence Appl Environ Microbiol 2001 67 2445 2452 10.1128/AEM.67.6.2445-2452.2001 11375149 PMC92893

[B27] Tanaka S Saito K Chon H Matsumura H Koga Y Takano K Kanaya S Crystallization and preliminary X-ray diffraction study of an active-site mutant of pro-Tk-Subtilisin from a hyperthermophilic archaeon Acta Crystallogr Sect F 2006 62 902 905 10.1107/S1744309106030454 PMC2242867 16946475

[B28] Gupta R Beg QK Lorenz P Bacterial alkaline proteases: molecular approaches and industrial applications Appl Microbiol Biotechnol 2002 59 15 32 10.1007/s00253-002-0975-y 12073127

[B29] Kumar CG Takagi H Microbial alkaline proteases: from a bioindustrial viewpoint Biotechnol Adv 1999 17 561 594 10.1016/S0734-9750(99)00027-0 14538129

[B30] Joo HS Kumar CG Park GC Paik SR Chang CS Bleach-resistant alkaline protease produced by a *Bacillus* sp. Isolated from the Korean polychaete, *Periserrula leucophryna* Process Biochem 2004 39 1441 1447 10.1016/S0032-9592(03)00260-7

[B31] Japanese Society of Medical Instrumentation Guideline for sterility assurance in healthcare setting 2010 Tokyo: Ministry of Health, Labour and Welfare, Japan

[B32] Otzen DE Protein unfolding in detergents: effect of micelle structure, ionic strength, pH, and temperature Biophys J 2002 83 2219 2230 10.1016/S0006-3495(02)73982-9 12324439 PMC1302310

